# A Double-Blind, Placebo Controlled, Randomized Trial to Assess the Impact of a Monthly Administration of 50,000 IU of Vitamin D3 for 6 Months on Serum Levels of 25-Hydroxyvitamin D in Healthy Young Adults

**DOI:** 10.1155/2013/652648

**Published:** 2013-11-13

**Authors:** E. Brunel, M. Schnitzler, M. Foidart-Dessalle, J. C. Souberbielle, E. Cavalier

**Affiliations:** ^1^Departments of General Medicine, University of Liege and Liege University Hospital, 4000 Liège, Belgium; ^2^Departments of Sport and Rehabilitation Sciences, University of Liege and Liege University Hospital, 4000 Liège, Belgium; ^3^Departments of Clinical Chemistry, University of Liege and Liege University Hospital, 4000 Liège, Belgium; ^4^Laboratoire d'Explorations Fonctionnelles, Hôpital Necker-Enfants Malades, 75014 Paris, France; ^5^Department of Clinical Chemistry, University of Liège, CHU Sart-Tilman, 4000 Liège, Belgium

## Abstract

In this double blind, unicentre, randomized, placebo controlled study, we evaluated the changes in 25-hydroxyvitamin D (25(OH)D) serum levels in 150 young Belgian adults (18–30 years), monthly supplemented with 50,000 IU of vitamin D (VTD) or placebo for 6 months, from November 2010 to May 2011. At T0, 30% of the population presented 25(OH)D serum levels below 20 ng/mL. In the VTD-treated group, mean serum levels increased from 21.2 ± 8.2 to 30.6 ± 8.8 ng/mL (*P* < 0.001) at T3mo and to 36.0 ± 9.2 ng/mL (*P* < 0.001) at T6mo. Despite documented VTD intake, no changes in serum levels were, however, observed in 10% of the treated group. In the placebo group, mean 25(OH)D serum levels decreased from 22.8 ± 8.5 to 14.0 ± 6.9 ng/mL at T3mo (*P* < 0.001) but returned to values not significantly different from those observed at T0 (23.5 ± 8.6 ng/mL) at T6mo. No difference between serum calcium levels was observed between the groups throughout the study. In conclusion, monthly supplementation with 50,000 UI of VTD in winter can warrant serum 25(OH)D levels above 20 ng/mL in 96.2% of those healthy young adults without inducing unacceptably high 25(OH)D concentration. This supplementation is safe and may be proposed without 25(OH)D testing.

## 1. Introduction

Vitamin D (VTD) deficiency is a worldwide problem [1, 2]. If all experts agree now to rely on serum 25-hydroxyvitamin D (25(OH)D) levels to estimate the VTD status, the 25(OH)D concentration defining VTD sufficiency is still a matter of debate, with recommendations that can significantly differ according to the experts. Indeed a recent report by the Institute of Medicine (IOM) indicates that a 25(OH)D level of 20 ng/mL is largely sufficient and the recommended dietary intake (RDIs) set forth (600 IU per day) should be sufficient for 97.5% of the population to achieve that level [3]. On the other hand, the Endocrine Society group considers that VTD deficiency corresponds to 25(OH)D levels of <20 ng/mL and insufficiency to levels of 20–30 ng/mL suggesting that higher intake than those recommended by the IOM is necessary to obtain an optimal VTD status [4]. If severe vitamin D deficiency, characterized by rickets in children and osteomalacia in adults, is a rare condition in developed countries, subclinical VTD deficiency/insufficiency is extremely frequent and can lead to parathyroid hormone (PTH) hypersecretion contributing to the development of osteoporosis in older adults [1]. Vitamin D deficiency is also associated with an increased risk of falls and fractures in the elderly [5, 6] and with potential “nonskeletal” effects, notably on the cardiovascular [7–9] and immune [10] systems as well as in cancers [11, 12]. Dietary sources of VTD are very limited and usual daily intakes are generally not higher than 200–400 IU in western countries [13–15]. The major source of VTD comes from UVB light-driven skin photosynthesis but, in northern latitudes like in Belgium (around 51° North), the UVB ray will be insufficient to allow the skin synthesis of VTD during approximately 6 months of the year. A pharmacological supplementation may thus be considered, at least during the winter months. In some patients such as those with osteoporosis, different supplementation schemes based on a baseline 25(OH)D measurements have been proposed with the aim to target a 25(OH)D level of 30 ng/mL or more [1, 11]. However, as 25(OH)D measurement is costly, it is suggested by many authors to supplement most patients and healthy persons without prior vitamin D testing [16]. For that purpose, one can rely on the recently published recommendations indicating that an intake of 600–800 IU/day [3] or 1500–2000 IU/day [4] is adequate. As observance of a daily supplementation is a critical issue in general, many doctors and patients prefer intermittent higher vitamin D3 (VTD3) doses given at intervals ranging from one week to one to four months. However, before proposing a systematic supplementation scheme with intermittent vitamin D doses during the “winter” months without prior 25(OH)D measurement, it is highly important to be sure that, with this scheme of supplementation, potentially toxic 25(OH)D levels are avoided, even transiently, and that serum calcium (CA) concentrations remain normal.

As the literature on the prevalence of VTD deficiency in the population of younger Belgian adults is scarce and as very few data regarding supplementation in this population are available, we aimed, in this prospective, randomized, placebo-controlled study, to measure the impact of a supplementation with 50,000 IU VTD3 per month during winter on the serum 25(OH)D levels in young (18–30 years) healthy volunteers. We also verified if that dose was sufficient to reach the IOM threshold for a healthy population (20 ng/mL), without any adverse clinical event or reaching levels that are considered as potentially harmful.

## 2. Methods

This study is a double blind, placebo controlled, randomized, comparative, and monocentre trial consisting in a supplementation of young healthy Caucasian volunteers every 4 weeks in winter (November 2010–May 2011), with either 50,000 IU VTD3 or placebo. 

In November 2011, after a screening visit of 195 candidates at the Liege University Hospital, 150 subjects who fulfilled the inclusion/exclusion criteria were randomly allocated to one of the treatment groups in a 1 : 1 ratio. They were healthy young males or females MD students or physical therapy students aged 18–30 years, with a body mass index between 18 and 30 kg/m^2^. Subjects having used within the last 30 days any over-the-counter medicinal products or herbal supplements or within the last 2 months food supplements containing VTD, those using UV light solarium, or patients treated with drugs interfering with VTD metabolism (phenytoin, phenobarbital, carbamazepine, glucocorticoids, digitalin, calcium, thiazides,…) were excluded from the study. Other exclusion criteria included hyperparathyroidism, past or current history of granulomatosis and especially sarcoidosis, urinary lithiasis, renal insufficiency, cardiac disease, cancer (of whatever origin), history of radiotherapy or chemotherapy, osteomalacia, human immunodeficiency virus or tuberculosis infection, acute or chronic alcohol, or drug abuse/dependence (including but not limited to cannabis) within 6 months prior to screening.

Study medication consisted in 1 mL ampoules containing each an oily solution of 25,000 IU/mL of cholecalciferol (D-cure, SMB Laboratories, Belgium). The placebo, also manufactured by SMB Laboratories, consisted of 1 mL ampoules containing the same excipient, but without cholecalciferol. The ampoules containing the placebo or VTD3 had the same appearance, color, and taste and were prepacked and consecutively numbered. Patients were allocated to one of the two groups (VTD or placebo) by one of the investigators (EC) without any contact with the subjects in order to have groups that were as much as possible similar in terms of age, hours of sport practice, and sex. Investigators (EB and MS) in contact with the volunteers were kept blind to group assignment. 

Six vials of study medication were dispensed at inclusion visit (T0) in November and in the control visit, in February (T3mo), after 90 ± 3 days. Two vials were orally absorbed in front of the investigators at these two visits. Written instructions to absorb 2 vials at 30 ± 3 days intervals were given at inclusion and control. An e-mail and a short message service (SMS) were sent to each participant at days 27,57,117, and 147 to ensure oral intake of the investigational drug at appropriate time. Compliance was further verified by systematic telephone interviews performed by a study nurse, to confirm the drug intake and register the vials numbers. Empty vials were returned to the investigator at the next visit (control visit in February and final visit in May). At each visit, all potential new complaints and symptoms (i.e., those not existing prior to signing informed consent) were recorded on the Adverse Event Clinical Record Form. Pre-existing complaints or symptoms that increased in intensity or frequency after having signed the Informed Consent Form were also registered as Adverse Events.

The study was approved by the Ethics Committee of the University Hospital of Liege and registered as Eudra-CT 2010-022454-17.

### 2.1. Laboratory Measurements

Blood samplings were performed in November (T0) before the first 50 000 IU dose was taken, in February (T3mo) just before the 4th dose, and in May (T6mo), one month after the 6th dose. 

We used the DiaSorin Liaison (Stillwater, MN, USA) for 25(OH)D and PTH (3rd generation) determination. Serum calcium was measured with the Roche Modular assay (reference range: 2.20–2.65 mmol/L).

All the samples were kept at −20°C before determination. Analyses were performed in batches. In our laboratory, 25(OH)D intra- and interassay coefficients of variation are <5% and <10%, respectively, and the functional sensitivity was found to be at 12 ng/mL. The laboratory participates in the DEQAS proficiency testing and is ISO 15189 accredited for 25(OH)D and Calcium determinations.

### 2.2. Statistical Analysis

Of the 195 screened volunteers, 150 were randomized and received at least one treatment or placebo during inclusion visit. One hundred and ten volunteers completed the trial and were included in the per protocol statistical analysis. Forty individuals were excluded because of documented major violations of the protocol (concomitant use of other sources of VTD in 3 instances or lack of adherence to the protocol in 37 cases (missed visit(s) or failure to take investigational medication)). 

After verifying that the 25(OH)D serum levels presented a normal distribution, an analysis of variance (ANOVA) was used for comparison of 25(OH)D levels at the different visits (November: inclusion; February: control, and May: final visit), in each group. A nonpaired student *t*-test was used for inter-groups comparison. Adverse events recording was obtained by questioning and examining subject at control and final visits. 

## 3. Results 

Baseline demographics and serum 25(OH)D levels are presented in Table 1.

No significant differences were observed at baseline between the groups. 

In November, 13% of this young Belgian population presented 25(OH)D levels above 30 ng/mL, approximately half of them had levels comprised between 20 and 30 ng/mL, while about 40% had values below 20 ng/mL (and 11% even <12 ng/mL). One individual presented a 25(OH)D concentration higher than 60 ng/mL (61 ng/mL). This subject was assigned to the VTD group.

At T3mo and T6mo, the 25(OH)D serum levels differed significantly between both groups (Table 2). After a 90-day treatment period (February), the mean serum levels increased from 21.2 ± 8.2 ng/mL to 30.6 ± 8.8 ng/mL in the VTD-treated group whereas it decreased from 22.8 ± 8.5 ng/mL to 14.0 ± 6.9 ng/mL in the placebo group. After 180 days (in May), the mean 25(OH)D serum levels reached 36.0 ± 9.2 ng/mL in the treated group while in the placebo group, mean values of 23.5 ± 8.6 ng/mL were recorded. 

**Figure 1:**
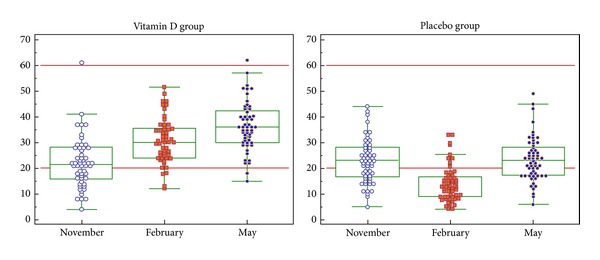
Serum 25(OH)D (ng/mL) levels observed in a young healthy population after 3 (February) and 6 (May) months of supplementation with a monthly dose of 50,000 IU of cholecalciferol or a placebo.

After 3 months of treatment with 50,000 IU of cholecalciferol, 8% of the subjects only still presented 25(OH)D levels <20 ng/mL and this percentage dropped to 4% after 6 months. None of the treated subjects did present 25(OH)D values that were still <12 ng/mL (Table 3). After the 6-month treatment, 70% and 96% of the subjects had a 25(OH)D concentration above 30 ng/mL and 20 ng/mL respectively. In the placebo group, 84.2% of the healthy volunteers had a 25(OH)D concentration <20 ng/mL in February (half of these subjects having a 25(OH)D <12 ng/mL). In May, the situation was very similar with the one observed in November: 33% presented 25(OH)D levels <20 ng/mL (5% <12 ng/mL) and 16% above 30 ng/mL. (Table 3). The distribution of the individual levels of 25(OH)D reached in the placebo and vitamin D is presented in Figure 1. The higher increases were observed in patients presenting the lowest initial 25(OH)D values, but those who had the higher 25(OH)D levels at inclusion were also those who had the highest values in May. In this study, body mass index (BMI) was not associated with the 25(OH)D increase during treatment with VTD3.

The highest serum 25(OH)D concentration (62 ng/mL) was observed in May in the treated group in a patient whose baseline level was 33 ng/mL. The patient (included in the VTD group) presenting 61 ng/mL at inclusion presented values at 31 and 57 ng/mL in February and May, respectively. 

The CA levels of the VTD and placebo groups were similar in November (2.43 ± 0.09 and 2.43 ± 0.08 mmol/L, resp.) and remained identical at T3mo and T6mo and none of the patients reported any sign or symptoms that could have been related with vitamin D intoxication nor presented CA values above 2.65 mmol/L. 

Finally, analysis of individual variations of 25(OH)D levels among the volunteers treated by cholecalciferol showed that 7 of them (13%) did not respond to the treatment as they failed to display a progressive increase in serum 25(OH)D levels despite regular and documented intake of the vitamin D doses.

## 4. Discussion

In this double-blind, placebo controlled, randomized trial, we assessed the impact of a monthly administration of 50,000 IU of vitamin D3 for 6 months on serum levels of 25(OH)D in 110 healthy young Belgian university students aged 18–30 years during winter months. 

Our results show that, in the middle of winter, 88% of our young and active subjects presented 25(OH)D values below 20 ng/mL, the threshold defined by the IOM as the limit of deficiency. If we have already shown the magnitude of this deficiency in our area in special populations like pregnant women [17] or elderly men living at home or in institutions [18], these data also show that the majority of these young active adults is also concerned by vitamin D deficiency, at least in winter. This can easily be understood as, in European countries with latitudes ≥37°N, photo-activation of previtamin D is minimal or absent between October and March and the dietary sources of vitamin D are scarce. In May, after an exceptional spring (actually, spring 2011 was the sunniest spring ever recorded in Belgium, with 707.15 hours of sunshine [19]), the distribution of 25(OH)D levels observed in these young healthy adults returned to the one observed in November. These low levels are generally associated with poorer outcomes (cardiovascular diseases, cancers,…) in different studies involving participants of older ages and only rarely concern adults of younger ages. However, low values of vitamin D have been associated in younger people with secondary hyperparathyroidism [15], respiratory tract infections [20], and multiple sclerosis [21]. A supplementation of this young population with vitamin D, at least in wintertime, may thus be of good practice. The IOM has stated that 600 IU could cover the needs of 97.5% of the population. In this study, we show that an intake corresponding to a daily dose of 1,666 IU was efficient to cover 96.2% of the needs of this young population after 6 months (92.4% after 3 months), a result close to the IOM expectancies, but with an extra 1,000 IU dose. It seems difficult to think that a supplementation with 600 IU would have reached this result in winter in our subjects, even if we did not test it. Accordingly, the IOM recommendation of 600 IU may be inadequate, at least in winter, to cover the needs of 97.5% of the population. 

This supplementation with 50,000 IU was safe as we did not observe any variation in calcium levels between the two groups. No complaints potentially linked to vitamin D toxicity have neither been registered by the physician who supervised the study and saw that patients at the different visits. The higher value achieved with this protocol was 62 ng/mL, a value that is far from the concentrations estimated as toxic (>150 ng/mL) [1] and is below the upper values observed in subjects living near the Equator and exposing themselves to the sun all year round [18]. 

Interestingly, we found that 4 participants did not respond to vitamin D supplementation as they did not increase their 25(OH)D levels in May. These patients might of course have been poorly compliants, even if we registered their intakes and if they confirmed their good compliance. They might also suffer from intestinal fat malabsorption, Crohn disease, or inflammatory bowel disease [22] even if the prevalence (13%) seems too high. This observation is interesting because it shows that the response to vitamin D is individual and can be affected by different reasons. 

This study has some limitations, like the rather small number of patients in each group, its monocentric design or the only one ethnicity tested. Nevertheless, it was conducted as a true randomized, placebo controlled trial as only one investigator, working in the laboratory without any contact with the patients had access to the results. Moreover, there is a limited number of studies involving young adults.

In conclusions we have shown here that the prevalence of vitamin D deficiency was very important in Belgian young adults in wintertime. A monthly dose of 50,000 IU of cholecalciferol from November to May was able to increase the 25(OH)D levels of 96.2% of this population above 20 ng/mL without inducing unacceptably high 25(OH)D concentration. Such a supplementation scheme was safe and may be proposed without 25(OH)D testing.

## Figures and Tables

**Table 1:** Baseline demographic data of the healthy young Belgian volunteers who agreed to participate in the study. Continuous variables are expressed as mean ± SD.

	Total	Placebo group	Vitamin D group
SEX:			
Male (*n*)	49	24	25
Female (*n*)	61	33	28
Age (years)	22.9 ± 2.5	23.0 ± 2.5	22.8 ± 2.6
BMI (kg/m²)	21.8 ± 2.1	21.9 ± 2.2	21.7 ± 2.0
25(OH)D (ng/mL)	22.0 ± 8.3	22.8 ± 8.5	21.2 ± 8.2
% subjects with 25(OH)D >30 ng/mL	12.7	14.0	11.3
% subjects with 25(OH)D between 20 and 30 ng/mL	48.2	49.1	47.2
% subjects with 25(OH)D between 12 and 19 ng/mL	28.2	26.3	30.2
% subjects with 25(OH)D <12 ng/mL	10.9	10.5	11.3
% subjects with 25(OH)D >60 ng/mL	0.9*	0	1.9*

*One individual.

**Table 2:** Serum 25(OH)D (ng/mL) levels observed in a young healthy population after 3 and 6 months of supplementation with a monthly dose of 50,000 IU of cholecalciferol or a placebo. The results are expressed as mean ± SD. A *P* value <0.05 is considered as significant.

	Placebo group	Vitamin D group	
Baseline (October)	22.8 ± 8.5	21.2 ± 8.2	NS
After 90 days (February)	14.0 ± 6.9	30.6 ± 8.8	*P* < 0.0001
After 180 days (May)	23.5 ± 8.6	36.0 ± 9.2	*P* < 0.0001
Intragroup comparison			
October—February	*P* < 0.0001	*P* < 0.0001	
February—May	*P* < 0.0001	*P* < 0.0001	
October—May	NS	*P* < 0.0001	

**Table 3:** Percentage of patients whose 25(OH)D levels were found in the <12, 20–30, and >30 ng/mL groups in the vitamin D or placebo group. One patient presented a 25(OH)D level >60 ng/mL in October and another one in May.

	Distribution of 25(OH)D serum levels					
	In volunteers receiving vitamin D Monthly doses of 50,000 IU of cholecalciferol			In volunteers receiving the placebo		
25-OH-Vit D	Baseline	After 90 days	After 180 days	Baseline	After 90 days	After 180 days
<12 ng/mL	11.3%	0%	0%	10.5%	42.1%	5.3%
12–19 ng/mL	30.2%	7.6%	3.8%	26.3%	42.1%	28.1%
20–29 ng/mL	47.2%	50.9%	26.4%	49.1%	12.3%	50.9%
≥30 ng/mL	11.3%	41.5%	69.8%	14.0%	3.5%	15.8%
